# Assessment of vitamin D deficiency in Qatar using Snibe-Maglumi X3 CLIA

**DOI:** 10.5339/qmj.2025.77

**Published:** 2025-09-15

**Authors:** Nouran Zein, Eman Al-Mohannadi, Asmaa Al-Ghanim, Nadin Younes, Salma Younes, Duaa Al-Sadeq, Shaden Abunasser, Parveen B Nizamuddin, Reem Al-Jehani, Maytha Al-Mohannadi, Gheyath Nasrallah

**Affiliations:** 1 Department of Biomedical Science, College of Health Sciences, Member of QU Health, Qatar University, Doha, P.O. Box 2713, Qatar; 2 Biomedical Research Center, Member of QU Health, Qatar University, Doha, P.O. Box 2713, Qatar *Email: gheyath.nasrallah@qu.edu.qa

**Keywords:** Qatar, vitamin D, prevalence, chemiluminescent, deficiency

## Abstract

**Background:**

Vitamin D deficiency is a widespread global public health concern. Although it is prevalent in the Middle East, available data remain limited. This gap in research leaves important questions unanswered regarding the exact prevalence of vitamin D deficiency and its potential impact on the population health. This study aims to determine the prevalence of vitamin D status among individuals in Qatar during the SARS-COV-2 pandemic (2020), using a fully automated chemiluminescent immunoassay.

**Methods:**

This epidemiological study included 1,000 participants selected from the Primary Health Care Corporation electronic medical record system. Random sampling was used to select the study sample, and data analysis was performed to determine prevalence by gender, age group, and patient’s continent of origin. Serum levels of 25-hydroxyvitamin D [25(OH)D] were interpreted according to the guidelines of The Endocrine Society. Levels were classified as follows: vitamin D deficiency (serum 25(OH)D<20 ng/mL), vitamin D insufficiency (20–29 ng/mL), optimal levels (30–50 ng/mL), and potential toxicity (>50 ng/mL).

**Results:**

The overall prevalence of vitamin D deficiency in Qatar was 49.8%. While there was no significant variation by gender, the prevalence varied significantly across age groups (*p* <0.0001) and by patients’ continent of origin (*p* <0.0001). Deficiency rates were highest in the 10–17 years age group (63%) and lowest among individuals aged >60 years (24.7%). Additionally, the deficiency rates were highest among individuals from Asian countries (51.6%) and the Middle East and North Africa (MENA) region (49.8%), and lowest among those from America and Europe.

**Conclusion:**

This study reveals a high prevalence of vitamin D deficiency in Qatar, affecting nearly half of the population and showing significant variations across age groups and geographical backgrounds. Individuals from Asian and MENA regions were more affected than those from America and Europe, highlighting possible lifestyle or genetic influences. These findings are intended to guide public health interventions and support global efforts to address vitamin D deficiencies.

## 1. INTRODUCTION

Vitamin D is an essential steroid hormone and nutrient, synthesized in the skin upon exposure to sunlight or obtained from dietary sources and supplements.^1^ It plays a vital role in maintaining overall health by regulating calcium metabolism, supporting immune function, and maintaining musculoskeletal integrity.^2,3^ Vitamin D deficiency can lead to various health issues, including osteoporosis,^4^ osteomalacia, rickets, mood disorders, and cardiovascular diseases.^5–7^

Although Qatar, located in the Arabian Peninsula, receives abundant year-round sunlight, its population encounters unique challenges in maintaining optimal vitamin D levels.^1,8^ Lifestyle factors such as indoor sedentary habits and traditional clothing practices may limit sun exposure among the population^9^, while dietary sources of vitamin D are often insufficient^10^, posing challenges to achieving optimal levels.^11,12^

Qatar has a distinct demographic composition, with approximately 88% of its population consisting of expatriates and males making up about 76.8% of the total population, largely due to labor migration in the construction and industrial sectors.^13^ The population is also relatively young and ethnically diverse, with approximately 69.9% aged between 25 and 54 years.^13^ These factors are likely to influence both sun exposure habits and dietary patterns, which are key to understanding vitamin D status.

The aim of this study is to evaluate the vitamin D status in Qatar in 2020 using the chemiluminescent immunoassay (CLIA) method. It provides an updated prevalence estimate of serum 25-hydroxyvitamin D [25(OH)D] levels across key demographic variables, including gender, age group, nationality, and patient’s continent of origin. These parameters were selected to identify population subgroups at higher risk of deficiency.

Our findings contribute to the limited but growing body of research on vitamin D status in the Middle East and aim to support public health decision-making. Additionally, this study enhances our understanding of how demographic and geographic factors intersect to influence vitamin D levels across diverse populations.

## 2. METHODS

### 2.1. Study setting

Qatar has experienced significant growth in recent years, supported by a robust healthcare system that ensures access to high-quality healthcare for both citizens and residents. The Primary HealthCare Corporation (PHCC) is the main publicly funded provider of primary healthcare services in Qatar, delivering its services through 28 health centers across the country.^14^ In addition, it maintains electronic medical records of all patients.

### 2.2. Study design and sampling strategy

This epidemiological study assesses the vitamin D status of the population in Qatar, providing an update based on samples collected in 2020. The study was approved by the Qatar University Institutional Review Board (QU-IRB 1469-E/21) and the PHCC Institutional Review Board (PHCCDCR202005047). For detailed information on sample collection, please refer to Syed et al. (2021).^15^ A total of 2,000 samples from individuals aged 10 years and above were extracted from the PHCC electronic medical record system; these samples had been previously used in a study,^15^ along with the demographic variables such as gender, age, nationality, and patient’s continent of origin. The study was conducted on 1,000 randomly selected samples from a total of 2,000 samples, including participants aged 10 years and above. Among them, 546 (54.6%) were male and 454 (45.4%) were female. A formal power analysis was not conducted prior to the study; however, a sample size of 1,000 is considered statistically adequate based on previous prevalence studies in comparable populations. Future research would benefit from conducting power calculations to optimize sample size and enhance statistical robustness.

This study was approved by the Qatar University Institutional Review Board (QU-IRB 282/2024-EM).

### 2.3. Data collection and laboratory procedures

Blood samples used in this study were collected from patients during a previous study.^15^ After collection, the samples were centrifuged to separate the serum and were then stored at −80°C in the Biomedical Research Center (BRC) laboratory at Qatar University until analysis. The samples were analyzed for vitamin D using the fully automated Maglumi^®^ chemiluminescence immunoassay system. Results were interpreted in accordance with The Endocrine Society’s guidelines,^16^ which classifying serum [25(OH)D] levels as follows: vitamin D deficiency (<20 ng/mL), vitamin D insufficiency (20–29 ng/mL), optimal vitamin D levels (≥30 ng/mL), and vitamin D toxicity (>50 ng/mL). Participants’ use of vitamin D supplements was not recorded, which limits the ability to account for potential confounding factors. Additionally, data on specific health conditions known to affect vitamin D metabolism (e.g. pregnancy, malabsorption syndromes, chronic kidney disease, osteomalacia) were unavailable, which may have influenced serum vitamin D levels in some individuals.

### 2.4. Measuring vitamin D by Snibe CLIA

At its core, CLIA functions on the principle of chemiluminescence. Essentially, when target analytes bind to specific antibodies, they initiate a cascade of enzymatic reactions that result in light emission. The intensity of the light emission is directly proportional to the concentration of the target analyte in the sample and can be accurately assessed using a specialized detection equipment.

The Maglumi^®^ X3 analyzer and Maglumi^®^ 25-OH vitamin D (CLIA) kit were used for the assessment of serum samples. According to the manufacturer’s instructions for Maglumi 25-OH Vitamin D (CLIA), a sample volume of 200 μL was used to measure vitamin D levels in the participants. Magnetic microbeads coated with an anti-25-OH vitamin D antibody were mixed and incubated at 37°C, then subjected to a wash cycle following magnetic precipitation. N-(4-Aminobutyl)-N-ethylisoluminol (ABEI), labeled with another anti-25-OH vitamin D antibody, is used to form sandwich complexes, followed by further incubation. The addition of starter reagents initiated a flash chemiluminescent reaction. The light signal was measured by a photomultiplier in relative light units (RLUs), which were proportional to the concentration of 25-OH vitamin D in the sample or controls.

The calculated coefficient of variation (CV%) was 6.2%, well within the acceptable intra-assay limit of 10%. We also evaluated the performance of liquid chromatography–tandem mass spectrometry (LC-MS/MS), which demonstrated reliable CV% values for 25(OH)2D2 and 25(OH)2D3, ranging from 15.5% to 3.6%, indicating consistent measurement precision (data not shown). Snibe’s limit of detection (LoD) was 0.5 ng/mL and its limit of quantification (LoQ) was 8 ng/mL. Prior validation studies have shown that this method exhibits high accuracy and reliability.^17,18^ However, additional local validation studies in Qatari populations could further strengthen its utility in regional diagnostics.

### 2.5. Quality assessment of the Snibe 25-(OH)-vitamin D CLIA

The Snibe CLIA was successfully validated in our laboratory (data not shown), where intra-assay CV% was assessed using seven quality control (QC) samples across multiple runs. The mean analyte concentration was calculated from multiple assay runs; additionally, the standard deviation (SD) was determined. Furthermore, the CV%, which measures relative variability in relation to the mean, was calculated by dividing the SD by the mean.

### 2.6. Statistical analysis

For the statistical analysis, we used a chi-square test to examine the association between the groups in our study. This test was chosen because it is appropriate for analyzing categorical data. To provide a deeper analysis of potential influencing factors, future iterations of this study may incorporate multivariate regression analysis to assess the impacts of continuous variables such as age or environmental exposure. Confidence intervals were included alongside *p*-values to enhance the interpretability and reliability of the findings. The analysis was performed using GraphPad Prism software (Boston, Massachusetts, USA), which enabled the calculation of the *p*-value. A threshold of 0.05 was used to determine statistical significance, with *p*-values below this cutoff indicating a meaningful association between the variables. This approach ensured that the results were both reliable and valid.

## 3. RESULTS

### 3.1. Vitamin D deficiency is highly prevalent in Qatar

Our study encompassed 1,000 participants, with 491 (49.1%) exhibiting deficient vitamin D levels and 266 (26.6%) displaying insufficiency, as shown in Table 1.

### 3.2. Age has a significant effect on vitamin D deficiency in Qatar

The study sample was divided into several age groups, as shown in Table 1. Among participants aged 10–17 years, 63% had deficient serum vitamin D levels. In the 18–39 years age group, 52.5% of the 440 participants were vitamin D deficient. Among the 306 participants aged 40–59 years, 41.8% had deficient serum vitamin D levels. Finally, in the ≥60 years age group, 24.7% of the 73 participants were vitamin D deficient. This difference across age groups was statistically significant, indicating that age is associated with the prevalence of vitamin D deficiency (*p* < 0.0001; refer Table 1).

### 3.3. Continent of origin has a significant effect on vitamin D deficiency in Qatar

The participants were categorized by region: Africa, Asia, Europe, MENA, and America. Among the 41 individuals from Africa, 43.9% were vitamin D deficient. Of the 285 participants from Asia, 51.6% were deficient. In Europe, 32.5% of the 40 participants were deficient, while another 32.5% had normal vitamin D levels. Among the 617 participants from the MENA region, 49.8% were deficient. Of the 17 participants from America, 35.3% were found to be vitamin D deficient. Overall, the differences in deficiency rates across regions were statistically significant (*p* < 0.0001; refer Table 1).

## 4. DISCUSSION

Vitamin D deficiency is a widespread public health concern globally. Although it is prevalent in the Middle East, published data from the region remain limited. This research gap leaves important questions unanswered regarding the prevalence and the potential impact of vitamin D deficiency on the population’s health. Our study aimed to assess serum vitamin D levels in an urban population. We found no statistically significant association between gender and vitamin D levels; however, age and continent of origin were statistically significant predictors of vitamin D status.

The absence of a significant gender difference in vitamin D levels contrasts with previous studies that reported such disparities.^19^ This discrepancy may be attributed to the diverse composition of Qatar’s population and the relatively balanced gender distribution in our cohort. Further research involving larger sample sizes and diverse populations may be needed to elucidate any potential gender-related disparities in vitamin D status.

In contrast, age showed a statistically significant association with vitamin D status, with younger individuals exhibiting higher deficiency rates. Consistent with previous research, this may be attributed to indoor lifestyles, prolonged screen time, and inadequate dietary habits.^20^ These findings highlight the need for targeted interventions among younger populations – particularly adolescents – to address emerging risk factors for vitamin D deficiency.

Continent of origin also played a significant role in vitamin D deficiency. Participants from Asia and the MENA region showed the highest deficiency rates, despite the availability of year-round sunlight in those regions. The majority of the participants were from the MENA region (*n* = 617), followed by Asia (*n* = 285), Africa (*n* = 41), Europe (*n* = 40), and America (*n* = 17). These findings are consistent with previous studies,^21,22^ and may be influenced by lifestyle factors, cultural clothing practices,^21,23^ and lower intake of vitamin D-rich foods.^24^ It also highlights broader socio-environmental and nutritional determinants of health among migrant groups.

Additionally, the study aimed to assess the prevalence of vitamin D deficiency in the population during the unprecedented circumstances of the COVID-19 lockdown in 2020. Compared to the Qatar Biobank (QBB) study in 2019, which reported an 88% deficiency rate, our 2020 study observed a significantly lower rate of 49%.^25^ This finding aligns with a study from Saudi Arabia, where 42% of the population exhibited either deficiency or insufficiency in 25(OH)D.^16^ This notable decrease in deficiency may reflect the impact of lifestyle changes during the COVID-19 lockdown: while some individuals spent more time indoors, others might have increased their sunlight exposure in private outdoor areas or improved their diets with more home-cooked meals. These behavioral shifts could have influenced vitamin D levels. However, as noted in the study’s limitations, we did not collect data on vitamin D supplementation, which may have also contributed to the improved levels.

The use of advanced techniques, such as the CLIA, enhanced the accuracy of our measurements. These findings highlight the importance of using high-precision analytical methods in population health studies. Understanding how age, geography, and lifestyle intersect in affecting vitamin D status is essential for designing effective public health strategies.

Our study has several limitations that should be considered. Non-normal distributions across age groups, continent of origin, and gender may introduce bias. Notably, we lacked data on participants’ use of vitamin D supplements, which may confound the observed outcomes – particularly during the pandemic period when supplement use increased. Additionally, the absence of data on vitamin D supplementation and relevant health conditions – such as pregnancy,^24^ malabsorption,^26^ kidney disease,^27^ or metabolic bone disorders – limits the interpretation of serum vitamin D levels across subgroups. These factors are known to influence vitamin D metabolism and can elevate the risk of vitamin D deficiency or insufficiency, and should be considered in future studies.

Moreover, sample collection during the COVID-19 pandemic may have introduced variables such as altered sunlight exposure, physical activity, diet, or supplement use – all of which can influence serum 25(OH)D levels. Although our findings indicate a lower prevalence of deficiency compared to pre-pandemic reports, the potential changes in behavior and healthcare access during this period should be considered.

While chi-square tests effectively identified statistically significant group differences, additional statistical modeling such as logistic regression could be employed in future research to control for confounding variables and better understand the influence of predictors like age, gender, and continent of origin. The inclusion of confidence intervals alongside p-values in this study further supports the reliability of our statistical conclusions.

Our findings also suggest that participants from Europe and America had higher proportions of normal vitamin D levels (32.5% and 35.2%, respectively) than those from the MENA and Asian regions. This may be explained by several factors, including increased skin exposure to sunlight due to cultural practices, outdoor lifestyle habits, and lighter skin tones, which allow more efficient synthesis of vitamin D. In contrast, individuals from regions such as the Middle East and Asia may have darker skin with higher melanin content, which naturally reduces the skin’s ability to produce vitamin D in response to UVB exposure. Furthermore, traditional clothing in these regions often covers most of the body, further limiting skin exposure to sunlight. These insights emphasize the importance of considering both environmental and biological factors when assessing vitamin D status in ethnically diverse populations.

A notable aspect of this study is its focus on vitamin D levels within the Qatari population. Despite the high prevalence of vitamin D deficiency in the region, comprehensive research on this issue has been limited. This investigation addresses a critical research gap by evaluating vitamin D levels within Qatar’s diverse population. While the 2019 QBB study focused primarily on Qatari nationals, our study includes both citizens and residents, providing a broader and more representative view of vitamin D status in the country. The inclusion of participants from various nationalities broadens the generalizability of our findings and supports cross-cultural insights into public health needs.

## 5. CONCLUSION

In conclusion, the use of the CLIA in our study enabled precise and reliable measurement of vitamin D levels, contributing to the robustness of our findings. This highlights the importance of employing advanced analytical methods for accurate assessment in both future research and clinical practice.

In summary, our study offers valuable insights into the dynamics of vitamin D status during the COVID-19 pandemic. We observed a vitamin D deficiency prevalence of 49.1% in 2020, which represents a substantial reduction compared to the 88% reported in the 2019 QBB study – a relative decrease of nearly 44%. These observed changes highlight the potential influence of lifestyle modifications induced by pandemic-related restrictions on nutritional status and emphasize the need for holistic approaches in public health interventions and policy formulation.

## AUTHORS’ CONTRIBUTIONS

GKN provided overall supervision and contributed to the study design and manuscript writing. NZ wrote the initial draft and conducted the data analysis. EA and AA performed the assays. DA contributed to manuscript preparation. All authors participated in manuscript writing and have reviewed and approved the final version of the manuscript.

## FUNDING

This work was supported by Grant No. UREP31-172-3-045 from the Qatar National Research Fund (a member of Qatar Foundation). All statements made in this work are the sole responsibility of the authors.

## ACKNOWLEDGMENT

We extend our sincere gratitude to the Primary Health Care Corporation and the Ministry of Public Health, Qatar, for providing the patient samples. We also wish to thank the GKN team for their constant support and invaluable guidance throughout the course of this project.

## CONFLICT OF INTEREST

The authors declare no conflict of interest.

## Figures and Tables

**Table 1.** Vitamin D status by gender, age group, and continent of origin among 1,000 randomly selected individuals (aged 10 years and above) from Primary Health Care Corporation records in Qatar during the COVID-19 pandemic (2020).

	Total participated	Normal	Insufficiency	Deficiency	Toxic	*p*-value
**Gender**						
Male	546	89 (16.3%)	154 (28.2%)	273 (50%)	30 (5.5%)	0.17
95% CI		4.5	7.7	13.7	1.5	
Female	454	88 (19.3%)	112 (24.6%)	218 (48%)	36 (7.9%)	
95% CI		4.4	5.6	10.9	1.8	
**Age (years)**						
10–17	181	19 (10.4%)	43 (23.8%)	114 (63%)	5 (2.8%)	**<0.0001**
95% CI		0.95	2.15	5.7	0.25	
18–39	440	77 (17.5%)	110 (25%)	231 (52.5%)	22 (5%)	
95% CI		3.85	5.5	11.6	1.1	
40–59	306	60 (19.6%)	88 (29%)	128 (41.8%)	30 (9.8%)	
95% CI		3	4.4	6.4	1.5	
≥ 60	73	21 (28.7%)	25 (34.2%)	18 (24.7%)	9 (12.3%)	
95% CI		1.05	1.25	0.9	0.45	
**Patient’s continent of origin**						
Africa	41	8 (19.5%)	11 (26.8%)	18 (43.9%)	4 (9.8%)	**<0.0001**
95% CI		0.4	0.5	0.9	0.2	
Asia	285	39 (13.6%)	83 (29.1%)	147 (51.6%)	16 (5.6%)	
95% CI		1.95	4.15	7.35	0.8	
America	17	6 (35.2%)	3 (17.6%)	6 (35.3%)	2 (11.8%)	
95% CI		0.3	0.15	0.3	0.1	
Europe	40	13 (32.5%)	11 (27.5%)	13 (32.5%)	3 (7.5%)	
95% CI		0.65	0.5	0.65	0.15	
MENA	617	111 (17.9%)	158 (25.6%)	307 (49.8%)	41 (6.6%)	
95% CI		5.6	7.9	15.3	2	
**Nationality**						
**Total**	1,000	177 (17.7%)	266 (26.6%)	491 (49.1%)	66 (6.6%)	

CI: Confidence interval, MENA: Middle East and North Africa. Bold *p* - value represents value <0.05, which means statistically significant.
